# Important considerations for feasibility studies in physical activity research involving persons with multiple sclerosis: a scoping systematic review and case study

**DOI:** 10.1186/s40814-017-0145-8

**Published:** 2017-06-09

**Authors:** Yvonne C. Learmonth, Robert W. Motl

**Affiliations:** 1 Department of Psychology and Exercise Science, Murdoch University, Murdoch, WA 6150 Australia; 2 School of Health Professions, University of Alabama at Birmingham, Birmingham, AL 35294 USA

**Keywords:** Feasibility studies, Scoping review, Feasibility metrics, Case study

## Abstract

**Background:**

Much research has been undertaken to establish the important benefits of physical activity in persons with multiple sclerosis (MS). There is disagreement regarding the strength of this research, perhaps because the majority of studies on physical activity and its benefits have not undergone initial and systematic feasibility testing. We aim to address the feasibility processes that have been examined within the context of physical activity interventions in MS.

**Method:**

A systematic scoping review was conducted based on a literature search of five databases to identify feasibility processes described in preliminary studies of physical activity in MS. We read and extracted methodology from each study based on the following feasibility metrics: process (e.g. recruitment), resource (e.g. monetary costs), management (e.g. personnel time requirements) and scientific outcomes (e.g. clinical/participant reported outcome measures). We illustrate the use of the four feasibility metrics within a randomised controlled trial of a home-based exercise intervention in persons with MS.

**Results:**

Twenty-five studies were identified. Resource feasibility (e.g. time and resources) and scientific outcomes feasibility (e.g. clinical outcomes) methodologies were applied and described in many studies; however, these metrics have not been systematically addressed. Metrics related to process feasibility (e.g. recruitment) and management feasibility (e.g. human and data management) are not well described within the literature. Our case study successfully enabled us to address the four feasibility metrics, and we provide new information on management feasibility (i.e. estimate data completeness and estimate data entry) and scientific outcomes feasibility (i.e. determining data collection materials appropriateness).

**Conclusion:**

Our review highlights the existing research and provides a case study which assesses important metrics of study feasibility. This review serves as a clarion call for feasibility trials that will substantially strengthen the foundation of research on exercise in MS.

## Background

Multiple sclerosis (MS) is a disease of the central nervous system characterised by inflammation, axonal demyelination and transection and neurodegeneration. The damage and its location within the CNS [1, 2] manifest as a loss of physical and psychological function, worsening of symptoms and reduction in quality of life (QOL). Over the past 20 years, there have been an increasing number of randomised controlled trials (RCTs) of exercise as a therapeutic intervention for managing the consequences of MS. There is substantial evidence from these RCTs for short-term benefits of exercise and physical activity amongst persons with MS who have mild-to-moderate disability [3–5]. Exercise is now considered one of the most important interventions for inclusion in the management of MS and its consequences [6].

Importantly, there is ongoing debate about the strength of the existing research regarding exercise and MS (e.g. [7] vs. [8]), and this is associated with limitations of previous RCTs. For example, there is limited evidence for the duration of benefits from participation in the exercise intervention after cessation of the intervention period [9–11]. Some data suggest that the benefits of exercise are not retained following cessation of an exercise programme [12]. The degree of benefits might depend on complying with the prescribed intervention [13], yet some exercise intervention studies report attrition rates of up to 42% [14]. There is substantial evidence indicating that persons with MS are not engaging in sufficient amounts of physical activity for accruing health benefits; this questions the broad translation of exercise benefits amongst persons with MS. The loss of benefits after cessation of exercise interventions and the poor uptake of physical activity amongst persons with MS is a public health concern and might be associated with the “bedrock” of previous research.

We believe that one major limitation of previous research that ultimately undermines efficacy, effectiveness and translation is that the majority of studies on physical activity and its benefits have not undergone initial and systematic feasibility testing (i.e. of the processes, resources, management and scientific outcomes of clinical trials). Instead, previous pilot studies have been undertaken in controlled environments (e.g. laboratory settings) to primarily demonstrate efficacy, but may not translate to or achieve real life effectiveness for continued, long-term uptake of physical activity [15]. They may have poor compliance with the intervention itself. This lack of systematic feasibility testing may result in the main study not achieving efficacy or effectiveness for changing the target outcome. This means that researchers may not be conducting the preliminary work that informs the design and implementation of an intervention before examining if the intervention has an effect on an outcome of interest. Essentially, we are forgoing building a strong and stable foundation that supports a robust framework and scaffolding for our house; without such a foundation, we might have a house of cards!

There is growing acceptance and recognition of the importance of conducting and reporting preliminary work on the feasibility of a study. Feasibility studies might be considered similar to phase 1 or phase 2 evaluation (or “proof of concept”) of pharmacological interventions [16]. However, it is acknowledged that interventions involving behavioural or lifestyle interventions (e.g. physical activity) may not fit into the classical clinical trial developmental model [17] (i.e. phase 1, evaluation of intervention safety, safe dosage range and identify side effects; phase 2, evaluation of whether the intervention is effective; phase 3, confirmation of intervention effectiveness, monitoring of side effects, comparison with common alternative treatments and safety; phase 4, after market evaluation of the intervention’s effect in various populations and determination of side effects associated with long-term use [17]).

**Fig. 1:**
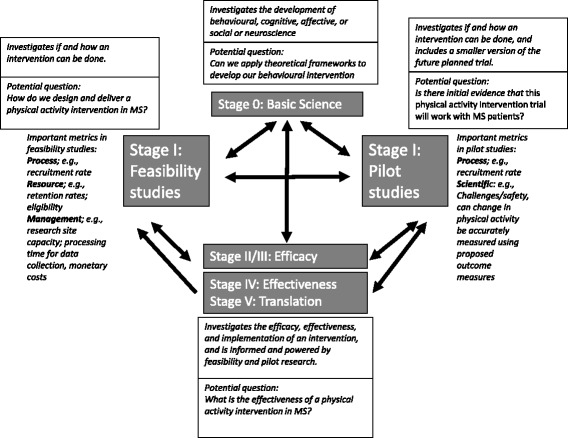
Relationship between feasibility, pilot and main trials

*Feasibility* is “an overarching concept for studies assessing whether a future study, project or development can be done” [18], and investigators attempt to answer a question about whether some element of the main study can and/or should be done, and, if so, how it could/should be done [16]. *Pilot studies* fit within the framework of feasibility [18], and investigators attempt to answer similar questions to a feasibility study and extend this to include a smaller scale version of the future study (i.e. a randomised or non-randomised trial). Feasibility studies of behavioural or lifestyle interventions (e.g. physical activity) fit within the stages of the updated National Institute of Health’s conceptual framework of intervention development [19], and these stages are outlined below and referred to in Fig. 1;

- Stage 0 includes the basic science of research and could include any behavioural, cognitive, affective or social or neuroscience being undertaken for development of the behavioural intervention.
- Stage I includes all activities related to the creation of a new intervention or the modification, adaption or refinement of an existing intervention. This stage includes feasibility and pilot testing.
- Stage II includes the testing of promising behavioural interventions in research settings, with research therapists/providers.
- Stage III includes the testing of intervention efficacy in a well-controlled, internally valid study in a *community setting* with *community therapists/providers,* and this includes community friendly fidelity monitoring and enhancement procedures as part of the intervention.
- Stage IV includes the testing of intervention effectiveness, and it examines behavioural intervention in community settings, with community therapists/providers, and maximises *external* validity.
- Stage V includes translation of the research. The translation involves implementing scientifically supported interventions into community settings and distributing intervention information and materials to the relevant groups [19].

Figure 1 contextualises the importance of feasibility studies within the larger process of developing and conducting clinical interventions. Stage I data is important to the advancement of stage II and stage III, while data from stage II to V can inform stage I for further refinement of the intervention [19]. For example, feasibility studies that target physical activity in a clinical population such as MS must consider metrics that are important for the success of future studies. These metrics provide information on if, and how, the research can be done in the first place. Further, stage I data will inform, and be informed by, the *Basic Science* data established in stage 0. Feasibility and pilot studies should be undertaken prior to the main stage II or stage III studies, [18], and we propose that information flow between these main stage II or stage III studies should inform alternate feasibility or pilot work; see Fig. 1. Such bidirectional informational flow will improve overall care for persons with MS and improve our understanding of clinical research in MS care.

Clinical journals are now accepting feasibility studies in recognition of the importance to the scientific field [20, 21]. Funding bodies recognise the importance of preliminary work and offer small grant programmes (e.g. the United States National Institute of Health Small Grant Program (RO3)). There are guidelines for conducting and reporting of preliminary studies [16, 22–24], and a framework has recently been proposed for defining feasibility and pilot studies [18].

Our objective is to investigate and clarify what feasibility processes have been investigated and described within the context of physical activity interventions in MS. We will undertake a systematic scoping review which will clarify the important definitions and conceptual boundaries of feasibility studies that provide context to the current position of preliminary research on this topic [25]. We provide examples of the process, resource, management and scientific metrics currently investigated in physical activity research in persons with MS and present the case that there are a number of important outcomes of feasibility that should be addressed in future research. We illustrate a case study of a pragmatic approach taken to assess the four feasibility metrics through a study of a physical activity and behavioural change intervention to increase the physical activity participation of persons with MS [26].

## Methods

Our literature review was informed by past reviews of preliminary studies in clinical research [18, 20, 27, 28] and was carried out in accordance with the Preferred Reporting Items for Systematic Reviews and Meta-analysis (PRISMA) [29]. Our inclusion criteria stipulated that papers had (1) to be published between 2000 and 2016, (2) to be written in English, (3) to study persons with MS, (4) to include interventions or assessments deemed to encapsulate physical activity and (5) to be indexed as a feasibility or pilot study. Additional exclusion criteria were (6) described as large scale (e.g. stage II or stage III studies), (7) discussion or review articles, (8) cross-sectional or retrospective data, (9) data gathering protocol only and (10) abstract only, and we further required that (11) the major consideration of the study was not to scientifically explore the treatment safety, dose, response and efficacy only (i.e. the effect of the intervention on the assessed outcomes). Our literature review was not designed to capture all preliminary studies in persons with MS that include aspects of physical activity; consistent with a scoping review [25, 30], it was designed to capture an overall summary of current research in this area that involves feasibility metrics.

We searched the following databases without date limits: PubMed, Science Direct, Scopus, SPORTDiscus and Web of Science. The key terms searched involved *Multiple Sclerosis*, acronyms of preliminary study (e.g. *Safety, Feasibility, Pilot, Preliminary, Phase 1, Stage 1)* and acronyms of physical rehabilitation (e.g. *Physical activity, Exercise, Rehabilitation)*. An example of our search strategy is provided as an Appendix.

Two reviewers read each study and determined examples of process, resource, management or scientific feasibility and used those to evoke discussion on current trends in preliminary studies involving persons with MS and that included aspects of physical activity. To ensure agreement, we used a checklist to identify examples of process, resource, management and scientific feasibility from previous reviews and discussion papers [16, 23, 27, 31, 32]. We met frequently to discuss study content and make a decision on the representation of process, resource, management or scientific feasibility.

Our four metrics of feasibility were derived from previous literature [23, 24] and are summarised in Table 1. When determining inclusion of process feasibility in the reviewed studies, we were interested in reporting of the *processes* that were keys to the success of the main study (i.e. where studies provided information on access to participants or ease of randomisation). For determining inclusion of *resource* feasibility in the reviewed studies, we were interested in reporting of the time and resource problems that may occur during the main study (i.e. participant retention, appropriateness of eligibility criteria, participation barriers, participant compliance with protocols, participant reaction to outcome assessment, equipment access and cost, intervention suitability within the proposed setting, staff training needs). For determining inclusion of *management* feasibility in the reviewed studies, we were interested in examining reporting of potential human and data management problems (i.e. research site capacity, equipment usage, data processing time, data completeness, data entry, software appropriateness, auditing of intervention delivery and ongoing ease of randomisation). For determining inclusion of *scientific* feasibility in the reviewed studies, we were interested in examining reporting of the safety, dose and response of the study to identify the appropriate intervention and assessment outcome. Examples of these include challenges noted by study personnel, appropriateness of data collection materials, potential threats to study validity, participant acceptance of the intervention, participant tolerance to the protocol, potential participant bias, data variability, treatment effect, consistency of results to expectations and appropriateness of participant group for receiving the intervention.

**Table 1:** Rationale for conducting a pilot/feasibility study

Metric and reason	Example of feasibility objectives in the literature	Summary of systematic review results	Result of feasibility outcome in Project GEMS [33]
Process: assesses the feasibility of the processes that are key to the success of the main study	Determine recruitment rates [23, 24] (e.g. response of participants to recruitment strategies, proportion of respondents who remain interested in study after information and screening)	Recruitment via MS Societies in the prospective location, clinician referrals, and trial awareness (through posters and leaflets) [7, 41, 44–46, 48, 52, 53, 55] Rationale for non-recruitment: travel difficulty, time restrictions [37]	Overall recruitment rate, 52% Successive recruitment via postal invitation, 25%; email invitation, 30%; and telephone invitation, 22%
	Determine ease of randomisation [24] (e.g. willingness of participants to be randomised to the proposed treatment group(s)	No examples	Not assessed
Resources: assesses the time and resource problems that can occur during the main study	Estimate retention of participants in the study [23, 24]^a^ (e.g. number of participants completing all aspects of study, number and reason for attrition)	All participants completed study [35, 37, 39, 41, 44, 47, 52] Drop out reasons: the intervention [7, 38, 58]; changes in time commitments [35, 51, 58]; unable to travel [42, 45, 46]; MS relapse [52]; other medical issues [7, 43, 45, 58]; non-compliance with the study protocol [7]; and lost contact [35, 37, 45]	90% of all participants completed study Drop out reasons: changes in time commitment and other medical issues
	Demonstrate appropriate eligibility criteria [23, 24]^a^ (e.g. are criteria too inclusive/exclusive)	All participants met inclusion criteria [47, 55] Exclusion reasons: participants were too active [35, 41]; too old [35]; recent relapse [35]; participation in another trial [35]; participation in formal rehabilitation [42]; non-MS diagnosis [35]; recent change in disease modifying therapy [35]; high fall history [41]; and cognitive deficits [41]	32% of interested parties did not meet inclusion criteria. Exclusion reasons; too active, low self-reported disability level
	Estimate barriers/refusals to participation [23, 24]^a^ (e.g. participant transportation problems)	Barriers identified: unable to travel [45, 46]; a change in personal time commitments [35, 51, 58]; MS relapse [52]; and other medical issues [7, 43, 45, 58]	10% of interested parties chose not to participate; unable to commit time
	Demonstrate compliance with study protocol [23, 24]^a^ (e.g. do participants adhere to correct dosage of intervention sessions)	Recorded via attendance at intervention [35, 37–40, 42–47, 50, 52, 58]; participant self-completed activity diaries [35, 37, 45–47]	75% of intervention participants were fully compliant with exercise sessions
	Demonstrate participants reaction to data collection and outcome assessments [23, 24]^a^ (e.g. participants understanding of data collection tools)	Compliance problems identified: participants unable to complete walking tasks [57]; general difficulties with assessment procedures [37]	Time to complete outcome questionnaires: baseline, 40 min; follow-up 48 min
	Estimate access to/cost of equipment, space, personnel time [24] (e.g. total cost of intervention delivery)	Cost identified: staff, equipment and facility overheads [54]	Cost per intervention participant: US$121.18
	Determine the suitability of the intervention in the proposed setting [24] (e.g. suitability of a home-based exercise programme)	Recorded via participant and staff interviews [43, 49, 52]	Feedback questionnaires and telephone interviews indicate intervention suitable
	Determine clinician training needs and competence [24] (e.g. training in outcome assessment and/or intervention delivery)	Recorded via reliability of assessor [44], Reported as staff training requirements described [42, 44, 55]	Not assessed
Management: assesses potential human and data management problems	Estimate research site capacity [24] (e.g. phone-line, database, clinic/research site capacity)	Reported as staff time required for recruitment [49]	Staff preparation and reporting time: 263 h across 4 staff members
	Estimate equipment usage [23] (e.g. ease of availability, personnel time, establishment of backup plan if equipment unavailable/broken)	Problems identified: equipment related data collection problems [51]	Not assessed
	Determine processing time for data collection [23, 24] (e.g. time to mail data collection materials, time to complete outcome assessment)	Reported as^a^ staff time required for equipment processing and preparation [51]	Mail turn-around-time to receive outcome assessments: 3 weeks
	Estimate data completeness [24] (e.g. missing data items, missing outcomes)	No examples	Missing data: 2.5% at baseline, 7.2% at follow-up
	Estimate data entry [24] (e.g. erroneous data)	No examples	Staff time to enter and check data: 61 h
	Determine software appropriateness for data [24] (e.g. capacity of software for data analysis)	Requirement identified: multiple software types necessary [55]	Not assessed
	Estimate processes to ensure and/or audit treatment fidelity [24] (e.g. clinicians adherence to protocol)	Reported as experienced staff providing feedback on intervention delivery to the intervention instructors [42]	Not assessed
Scientific: assesses the safety, burden data collection and response to the study	Estimate challenges perceived/experienced by study personnel [23] (e.g. skills required to use assessment protocol)	Requirement for more time for participants to complete assessments [49]	Not assessed
	Determine data collection materials appropriateness [23] (e.g. user friendly for data collection personnel)	No examples	Participants commented the outcome assessments were burdensome
	Demonstrate potential extraneous variables which may threaten the validity of the research [24] (e.g. participant’ prior knowledge of intervention content)	No examples	Not assessed
	Determine the acceptability to participants of the intervention(s) [24] (e.g. participants view on intervention before/during/after)	Refer to original publications for individual details of the most acceptable exercise intervention [35, 37, 56, 57]	Positive written and verbal feedback from participants on the appropriateness of the intervention
	Estimate data variability in controlled trials [23, 24] (e.g. statistical analysis performed to establish baseline differences between groups)	No significant baseline differences [7, 41, 45–49, 58]	No significant baseline differences in demographic or clinical metrics
	Estimate treatment effect [23] (e.g. effect of primary outcome—not recommended)	Significant interaction—recommend primary outcome [37, 39, 45, 46, 52, 58] No significant interaction—cannot recommend primary outcome [44, 48, 49]	Significant interaction—recommend primary outcome
	Determine appropriateness of target group for intervention [24] (e.g. are participants receptive to change expected in intervention)	Positive feedback from participants on the appropriateness of the intervention [56]	Positive written and verbal feedback from participants on the appropriateness of the intervention

^a^Considered assessment of process in reference [24]

We initially planned to exclude all pilot studies that primarily reported upon the scientific outcome of treatment effect of studies, but, during our review, we established that other feasibility metrics were investigated in pilot studies, and we therefore chose to not exclude the studies.

### Case study

Our literature review highlighted a need to design a pragmatic study to address feasibility in exercise-based physical activity research within an MS population. We designed a study entitled *Guidelines for Exercise in Multiple Sclerosis (GEMS),* and this represents our pragmatic case study. The GEMS project [26, 33] is part of a larger research agenda to understand and improve exercise behaviour in persons with MS and received ethical approval from a Midwest, USA University ethical review board. The randomised controlled study examined the feasibility of a 4-month home-based exercise training programme designed based on recent physical activity guidelines for MS and supplemented by behavioural strategies (e.g. video coaching calls) for compliance. The study was undertaken from summer 2015 until spring 2016, and we published our study design and methods for the project [26] and results of the study [33]. Overall, the study was a 4-month home-based exercise training programme designed based on recent physical activity guidelines for MS and supplemented by behavioural strategies for compliance. Participants with mild-to-moderate MS were recruited and randomised into an intervention or wait-list control condition. Intervention participants received exercise equipment and received compliance materials and coaching calls. All participants completed relevant validated outcomes (e.g. exercise participation). Intervention participants provided feedback following the intervention via a written feedback survey and telephone interview.

We established clear data sources and outcome variables to assess each feasibility metric. Full details of all monitoring and assessment strategies, data source and outcome variables for areas of process, resource, management and scientific feasibility can be assessed in our publications [26, 33].

## Results

**Fig. 2:**
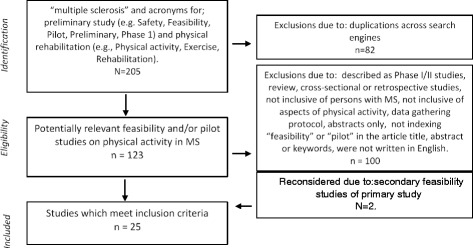
PRISMA flow chart of literature search

Our literature search identified 205 studies (Fig. 2), and 82 were duplicates across search engines. We initially excluded 100 studies for the following reasons: described as large scale (i.e. stage II or stage II studies) (*n* = 4), discussion or review papers (*n* = 21), cross-sectional (*n* = 2) or retrospective (*n* = 2) studies; not inclusive of persons with MS (*n* = 4); did not included aspects of physical activity (*n* = 33); were data gathering protocols (*n* = 4); were abstracts only (*n* = 26); did not use the terms “feasibility” or “pilot” in the article title, abstract or keywords (*n* = 2) and were not written in English (*n* = 2). Two articles initially excluded due to not including the terms pilot or feasibility were reconsidered as these were secondary publications of a feasibility study.

There were 25 studies deemed of interest to inform the current status quo of preliminary physical activity studies in MS population. We read these studies and undertook a content analysis focused on our four feasibility metrics of interest, *process, resource, management and scientific* feasibility. Table 1 provides a summary of the four feasibility metrics, data which can be established during feasibility studies and examples of outcomes used to gather data. Table 1 further provides the detailed results of our feasibility case study.

Of the 25 articles, 21 included an intervention [7, 34–53], 1 compared recruitment methods into an intervention study [53] and 1 was a cost analysis of the same intervention study [54]. One study was an investigation into the feasibility of an outcome assessment [55], and one was a qualitative analysis of participant opinions following an intervention [56].

The words feasibility and pilot were used in the title of 6 [35, 37, 47, 48, 51, 52] and 8 [7, 34, 39–42, 44, 51] studies, respectively, and 23 [7, 35–54, 57] studies indexed the word feasibility in the abstract, and 7 [7, 35, 40–43, 48] studies included pilot in the abstract. One [35] study included feasibility in the key words, and one [52] study included pilot in the key words.

The reporting mechanism and a summary of results for each of the four feasibility domains are detailed in Table 1. We identified that aspects of process feasibility were included in 10 studies, aspects of resource feasibility were discussed in 21 studies, aspects of management feasibility were included in 4 studies and aspects of scientific outcome feasibility were included in 16 studies. Within our discussion of how past studies assessed the four feasibility domains, we further provided abbreviated results for these domains as assessed in our case study, Project GEMS [33].

### Process feasibility

Practical aspects of recruiting participants were reported in 9 studies [7, 41, 44–46, 48, 52, 53, 55]; in the one study that compared recruitment strategies, researcher established that MS clinic recruitment (compared with consultant referral and trial awareness) was successful. In the Project GEMS case study, we took a successive recruitment approach (i.e. postal, email and then telephone invitation) to yield a 52% recruitment rate. No study discussed the willingness of participants to be randomised to either the main intervention group or the comparison group, and we did not include this aspect in our case study.

### Resource feasibility

The retention of participants in the studies was reported in 17 studies [7, 35, 37–40, 43–48, 50–52, 55, 58], and some studies discussed participant attrition. Reasons for study attrition included the following: aspects of the intervention, changes in time commitments, unable to travel, MS relapse, other medical issues, non-compliance with the study protocol and lost contact [35, 37, 45]. In our case study, we reported high numbers of participants completing Project GEMS and identified that changes in time commitment and other medical issues resulted in participant attrition. Eligibility criteria were reported in 7 studies [7, 35, 41, 42, 44, 47, 48, 55], and reasons for not meeting the eligibility criteria included the following: potential participants were too active, too old, had a recent relapse, were participating in another trial, were participating in formal rehabilitation, had a non-MS diagnosis, had recent change in disease modifying therapy, had a high fall history and had cognitive deficits. In Project GEMS, potential participants were excluded due to being too active or having a disability level lower than our criteria. To estimate barriers to participation, data on retention were considered in 8 studies [7, 35, 43, 45, 46, 51, 52, 58], and barriers included are being unable to travel, unable to commit time, MS relapse and other medical issues. In Project GEMS, the only identified barrier to participation was from potential participants unable to commit time. Compliance with the intervention was demonstrated in 13 studies via participant attendance at intervention sessions [35, 37–40, 42–47, 50, 52, 58], and in our case study, we also reported compliance via the number of completed exercise sessions. Participants reaction to outcome assessments were identified in 2 studies [49, 56], and issues identified included participants being unable to complete walking-based outcome assessments. In Project GEMS, we determined participant reaction to outcome assessment via the time it took for completion of the outcome assessments, and we gathered feedback comments from participants on the topic of completing outcome assessments. One reviewed study identified study costs based upon staff, equipment and facility overheads. In project GEMS, we calculated cost based upon research overheads and participants remuneration. The intervention suitability was determined in 3 studies using staff and participant interviews [42, 44, 55], and we used similar methodology in Project GEMS. In Project GEMS, positive feedback responses indicated that the intervention was suitable. Aspects related to clinician training need and competence were identified in 3 studies [42, 44, 55], and we did not include this aspect in our own study.

### Management feasibility

Research site capacity was identified in 1 study [53], and this was through the length of time staff devoted to recruiting participants to the study. In our case study, we reported staff time requirements. One study established data on equipment usage [55], and this was by identifying problems with the data collection equipment; we did not assess this metric in Project GEMS. One study identified processing time for data collection and preparation [51], and in Project GEMS, we assessed this metric using the mail turn-around-time for us to receive outcome assessments back from participants. We could not identify any past study which provided examples of data completeness. In Project GEMS, we did assess this metric and we did so via the percentage of missing data. Similarly, we could not identify any past study which estimated data entry, and in Project GEMS, we assessed this metric by identifying the time for staff to enter and check data. One study identified software appropriateness for data entry [55], and another study used procedures to audit treatment fidelity [42]; we did not assess either of these metrics in Project GEMS,

### Scientific feasibility

The challenges experienced by study personnel were reported in 1 study [49], where it was highlighted that more time was required for participants to complete assessments. We did not assess this metric as part of Project GEMS. We did not identify any past study which reported on the appropriateness of data collection materials; in Project GEMS, feedback from participants indicated that the outcome assessments (e.g., data collection materials) were burdensome. There were no examples from the literature which examined potential extraneous variables which may threaten the validity of the research, and we did not assess this metric in our study. Four studies assessed the acceptability of the intervention [35, 37, 56, 57], and this was by identifying what was the most commonly chosen form of exercise. In Project GEMS, we identified from written and verbal feedback that overall, the intervention was acceptable to all participants. Eleven randomised controlled studies estimated data variability in controlled trials [7, 41, 45–49, 58], and it was found that there were no significant differences in outcome measures at baseline. In Project GEMS, we identified no significant differences between outcome measures at baseline. Nine studies reported what the effect of the intervention was on their primary outcome measure [37, 39, 45, 46, 52, 58]; in Project GEMS, we reported the effect of the intervention on our primary outcome measure. Finally, 1 study determined the appropriateness of the target group for receiving the intervention [56], and in Project GEMS, we established that the intervention was appropriate for our written and verbal feedback.

## Discussion

Our discussion of the literature highlights that important areas of feasibility are not being adequately addressed in preliminary physical activity studies in persons with MS and this has major implications for the strength of the existing research regarding exercise and MS. We believe this might be addressed in future research through the provision of this scoping review of the literature. For example, process feasibility (e.g. recruitment) was discussed in less than half of the studies, and there is a need for more information on participant reactions to randomisation; it is not clear whether randomisation during clinical trials affects recruitment rates and how this might inform trial design (e.g. preference designs vs. RCTs). Metrics related to resources (e.g. time and resources) were explored in many studies, and this suggests that researchers are efficient at reporting these methodological areas. However, there is still a need for clarification of some aspects; for example, we do not know the costs involved in undertaking physical activity research programmes and these data are essential for both realistic application to funding bodies and the application of the intervention in everyday clinical practice. Data on management feasibility are not well reported in the included literature; without these data, replication of research studies and application of the intervention in clinical practice may be carried out in lieu of full knowledge regarding staffing and infrastructural requirements, and this may ultimately lead to failure of the intervention. Finally, there are many important aspects of scientific outcome feasibility, which require further explanation in preliminary studies, the reporting of intervention problems, and participant feedback will arm researchers with important knowledge to improve the science of physical activity interventions for persons with MS. There was a limitation of some studies to report hypothesis testing for baseline comparisons and the direct calculation of treatment effects, however these areas are not recommended in the new CONSORT extension guidelines [16].

Our case study, Projects GEMS, enabled us to systematically record feasibility study in the domains of process (e.g. recruitment), resource (e.g. communication with participants), management (e.g. missing data items) and scientific outcomes (e.g. compliance during the intervention and participant feedback). This approach allowed us to record challenges to the design, methods and procedures of the study. We designed the GEMS project to be consistent with guidelines for the conduction and reporting of preliminary studies [22–24]; however, we acknowledge that not all aspects of feasibility study were assessed during the GEMS project. Importantly, there are areas of feasibility such as determining ease of randomisation and providing details of variables which may threaten the validity of the research which are still to be discussed in the physical activity in persons with MS literature. We encourage researchers to monitor the four domains of feasibility when first designing behavioural interventions in persons with MS and to use these strong foundations to build improved interventions and better current research design.

## Conclusions

The strength of the evidence indicating that exercise is beneficial for persons with MS is growing; however, the evidence is not yet unequivocal as there are some examples of exercise interventions being ineffective in improving the primary outcome in comparison with a control group, e.g. [7, 59], and/or exercise behaviour, potentially, being difficult to sustain [9–11]. It is of importance that we are not yet designing high impact studies using effective and sustainable interventions, and as a result, we are not articulating the exercise message clearly to the wider MS community. We believe that this can be addressed through feasibility research, and there is lack of a systematic approach for such trials in exercise and MS. Our review highlights the existing research and our recent study, and we hope that this serves as a clarion call for feasibility trials that will substantially strengthen the foundation or bed rock of research on exercise in MS. The time is ripe, and we invite your participation on feasibility research on exercise in MS.

## Appendix

Example search strategy; PubMed search

(“Safety” OR “Efficacy” OR “Feasibility” OR “Pilot”, OR “Stage 1” OR “Stage one”) AND (“Physical activity” OR “Exercise”) AND (“Multiple Sclerosis*”).
